# Dietary Interventions Modulate Cell Competition and Locomotor Decline in an Alzheimer’s Disease *Drosophila* Model

**DOI:** 10.3390/cells14242011

**Published:** 2025-12-17

**Authors:** Carolina Costa-Rodrigues, Jovin R. Jacobs, Joana Couceiro, Catarina Brás-Pereira, Eduardo Moreno

**Affiliations:** 1 Champalimaud Centre for the Unknown, Av. Brasília, 1400-038 Lisbon, Portugalcatarina.pereira@gimm.pt (C.B.-P.); 2 Department of Neurobiology and Anatomy, University of Utah, Salt Lake City, UT 84132, USA; 3 Gulbenkian Institute for Molecular Medicine, Rua da Quinta Grande 6, 2780-156 Oeiras, Portugal

**Keywords:** Alzheimer’s Disease, nutrition, cell competition, *Drosophila*, *azot*

## Abstract

**Highlights:**

**What are the main findings?**

- Cell competition timing and efficiency, together with locomotion, can be modulated by dietary regimen, in a *Drosophila* Alzheimer’s Disease model
- Yeast-based diet potentiates Amyloid-β42 accumulation, triggering cell competition and locomotion decline
- Tissue fitness and diet can synergistically modulate the strength and profile of neuronal cell competition

**What are the implications of the main findings?**

- Targeting neuronal cell competition through nutritional interventions may offer new avenues to modulate the course of neurodegeneration
- Conserved fitness-sensing pathways like Flower can provide tractable biomarkers and therapeutic approaches for neurodegenerative diseases

**Abstract:**

Alzheimer’s Disease (AD) is a neurodegenerative disorder characterised by Amyloid-beta 42 (Aβ42) plaque accumulation and cognitive decline, with current treatments focused on symptomatic relief. Emerging therapeutics, such as dietary interventions, can modulate cognitive decline and delay AD progression. Our previous work in *Drosophila melanogaster* identified cell competition as a key mechanism that eliminates unfit neurons in an AD model, improving locomotion by removing the unfit neurons expressing *flower^LoseB^* and *ahuizotl* (*azot*). Here, we explored how diet influences *azot*-dependent cell competition and locomotion in the AD model. Flies were fed with either a yeast-based diet (YBD) or a synthetic (SAA) diet for up to 28 days. In contrast to YBD, SAA delayed cell competition activation until day 21, coinciding with locomotion improvement and delayed Aβ formation. The overexpression of the *human Flower (hFWE)* isoforms in a *Drosophila* neuronal context revealed functional conservation: *hFWE1* acted as the sole loser isoform, and *hFWE2* as a winner isoform. With the YBD, forcing cell competition by expressing *hFWE2* in the AD model led to an accumulation of unfit cells and promoted worse locomotion phenotypes over time compared to with the SAA diet. Our data highlights the complex interaction between diet, cell competition, and Aβ toxicity, offering new therapeutic insights.

## 1. Introduction

The prevalence of neurodegenerative diseases (NDDs) is rising significantly among the elderly. Dementia impacts over 55.2 million people worldwide, and projections show that will grow to up to 78 million by 2030, according to the World Alzheimer Report 2021 [1]. Alzheimer’s Disease (AD) has a multifactorial nature, which involves neuronal death; the extracellular accumulation of Amyloid-β (Aβ), mainly the Aβ42 peptide aggregates; and impaired insulin signalling [2,3,4]. Patients experience cognitive decline, memory loss, and behavioural impairments due to the loss of neuronal processes and aberrant network activity [5,6].

NDDs are complex due to human intricacy, and several hypotheses have been proposed as the cause of AD, yet a unified theory remains to be elucidated [7]. *Drosophila melanogaster* has emerged as a key model for NDD research [8], given its specific characteristics such as a short life cycle, ample progeny, and conservation of fundamental cellular processes and signalling pathways, including nutrient-sensing pathways [9,10,11]. Flies possess a simpler nervous system than humans, which has a blood–brain barrier and is composed of the same cell types, including neurons and glia [9,10]. They can also execute complex motor behaviours and memory and learning assays [12,13]. These characteristics make *Drosophila* a valuable model organism for studying NDDs.

Despite extensive research, AD treatments remain ineffective and often focus on symptoms rather than halting disease progression [14]. Recent studies link cell competition to AD, showing that removing unfit neurons restores locomotion in flies [13]. Cell fitness is both relative and context-dependent, as it varies according to neighbouring cells, and a fit cell in one context might be unfit in another [15,16]. When differences in fitness emerge within tissue, the cell competition mechanism ensures the removal of less-fit cells, thereby maintaining tissue and organismal homeostasis, in both invertebrates and vertebrates [17,18,19,20]. Tissues rely on different mechanisms of cell competition to eliminate unfit cells; for example, the fitness fingerprint-mediated cell competition mechanism was described by our group to be present in an AD context [13,21,22]. Flower (Fwe) isoforms are localised at the membrane, with the *fwe^Ubi^* isoform expressed in fitter winner cells, while the *fwe^LoseA/B^* isoforms are expressed in less-fit loser cells [13,16,23,24]. The Fwe code is cell-type specific and *fwe^LoseA/B^* triggers cell elimination in epithelia, whereas in neuronal cells, only *fwe^LoseB^* is sufficient to induce cell elimination [25]. The activation of the fitness sensor, *ahuizotl* (*azot*), occurs downstream of *fwe^Lose^*, being required for apoptosis induction through the activation of the pro-apoptotic gene *hid* [26]. The loss of *azot* impairs cell competition, reducing lifespan and tissue regeneration [13,26]. Variations in *azot* expression in the gut also modulate lifespan, confirming its importance in ageing [27]. Recent studies propose that less than 50% of *fwe^Lose^*-positive cells express *azot* and die, and in *azot^−/−^* flies, loser cells are still eliminated, revealing the existence of an *azot*-independent elimination mechanism [28]. Additionally, *azot*-positive cells can persist in the tissue without triggering apoptosis, which suggests that additional checkpoints downstream of *azot* exist prior to cell elimination [28]. Despite our limited knowledge of the *fwe/azot* pathway, the *Drosophila* homolog of the SPARC/Osteonectin family, Sparc, is known to be upregulated in loser cells, protecting these cells from elimination in a cell competition-specific manner, and counteracting the effect of Flower [23]. Furthermore, Sparc and *Fwe* pathways are independent and act in parallel, and *azot* is responsible for integrating the signal of both pathways [23].

Diverse mice and fly models have been used to study AD (reviewed in [11]). One of those *Drosophila* models was developed by Casas-Tinto et al. (2011) and is characterised by the overexpression of the human Amyloid-β 1–42 (*hAβ42*) in the developing retina using the Glass Multiple Repeat (GMR) driver (GMR-Gal4). The two copies of *hAβ42* (2x *hAβ42*) carry a secretion signal peptide (UAS-2x hAβ42), ensuring its secretion to the extracellular space, promoting random distribution of the plaques extracellularly [29]. This approach mimics the Amyloid Precursor protein (APP) duplication linked to early-onset familial AD and induces high levels of *hAβ42,* promoting a strong phenotype, and eyes with small and disorganised retinas [29]. In flies that are 20 days old, the retinas are more disorganised and vacuolated than those of 1-day-old flies, showing increasing signs of neurodegeneration as flies age [29]. Coelho and colleagues demonstrated that in *Drosophila*, the neurons near the hAβ42 plaques express *flower^LoseB^*, tagging them for elimination through *azot* expression [13]. Furthermore, flies with hAβ42 plaques had motor impairments similar to AD patients, and the elimination of these loser neurons was sufficient to restore motor coordination and memory formation [13]. Moreover, downregulating *azot* intensified locomotion impairments, while an extra copy of *azot* enhanced competition and improved motor behaviour. These findings suggest that *azot* activation may help counteract AD-related motor decline [13].

Recent developments have highlighted dietary and lifestyle changes as an approach to slow AD progression and cognitive decline [30]. Research shows that a Mediterranean diet and diets improving metabolic syndrome phenotypes are key strategies to tackle NDDs [31]. Despite recent studies revealing diet’s influence on AD, further research is needed to fully understand it. Some reports show that diet can also modulate cell competition mechanisms in a cancer context. For instance a high-fat diet decreases the apical elimination of Ras^V12^-transformed cells from mice intestinal and pancreatic epithelia, impairing cell competition [32]. A high-sugar diet promotes tumour growth and metastasis in *Drosophila* by transforming Ras/Src-induced tissue growth into aggressive and metastatic tumours, with cells escaping cell competition [33]. Hamann et al. (2017) showed that glucose withdrawal induces entosis, allowing winner cells to obtain nutrients [34]. Additionally, mTOR signalling was shown to act as a fitness sensor, as unfit cells exhibited decreased mTOR activity, indicating the involvement of nutrient-sensing pathways in cell competition [35]. Hyperinsulinemia promoted tumour growth, allowing cells to escape elimination due to increased protein synthesis [36]. These findings show the importance of nutrient-sensing pathways in cell competition and how diet modulates cell competition in tumoural contexts, opening up new possibilities for therapeutics involving cell competition mechanisms. However, there are currently no studies directly assessing the interplay between diet, AD, and cell competition mechanisms. Therefore, we aimed to understand how diet influences *azot*-dependent cell competition mechanisms and their implications in a *Drosophila* Alzheimer’s Disease model.

Our results show that diet modulates *azot*-dependent cell competition mechanisms in *hAβ4*-expressing flies. The activation of cell competition at 21 days improved locomotion when flies were fed with a synthetic (SAA) diet, but not in flies fed with a yeast-based diet (YBD). SAA diet delayed the rise in hAβ42 levels, allowing flies to be healthier for longer periods. In contrast, when *hAβ4*-expressing flies were fed with a YBD, cell competition was induced earlier at 14 days, as previously shown by Coelho et al. (2018) [13], but flies experienced a gradual locomotion decline, possibly from impairments to unfit cell elimination downstream of *azot*. Therefore, SAA seems to be more beneficial than the YBD for *hAβ4*-expressing flies. Our data suggests that diet can regulate the *hAβ4*2 toxicity by delaying the accumulation of toxic aggregates and promoting the elimination of unfit cells. Thus, it provides a foundation for identifying therapeutic approaches to manipulate fitness-fingerprint-mediated cell competition to treat age-related diseases.

## 2. Methods

### 2.1. Drosophila Husbandry and Stocks

*Drosophila melanogaster* stocks were maintained at 25 °C, with 60% humidity and a 12 h light/12 h dark cycle. Flies were kept in vials with a yeast-based diet (YBD Recipe) containing per 1 L: 80 g of Barley Malt SyrupPróvida, Portugal, 22 g of Beet syrup Grafschafter, Germany, 80 g corn flour, 18 g of Instant yeast Lesaffre, Portugal, 10 g Soy flour Centazzi, Portugal), 8 g of AgarNZYtech, Portugal, 12 mL Nipagin 15%, and 8 mL Propionic acid > 99% (Acros, Portugal).

The stocks used were *UAS-2x-hAb42* [29] *UAS-CD8:GFP*, *UAS-nls:LacZ*, UAS-*fwe^LoseB^* [24] *UAS-hFWE1* (this study), *UAS-hFWE2* (this study), *UAS-hFWE3* (this study), *UAS-hFWE4* (this study), *azot::mCherry* [13], *UAS-dsfoxo* (VDRC #107786), *UAS-akt* (BDSC #8191), and *GMR-Gal4* (BDSC #1104).

To perform the diet studies, we used *GMR-GAL4*,*UAS-2xhAβ42* generated by Sergio Casas-Tinto [29] for the AD model, and we recombined GMR-Gal4 with *UAS-CD8:GFP* to establish the control model (healthy flies). Flies from AD model [*y- w- hsFLP; GMR-GAL4*,*UAS-2xhAβ42/CyO; azot::mCherry/TM6B*] or control model [*y- w- hsFLP; GMR-GAL4*,*UAS-CD8:GFP/CyO; azot::mCherry/TM6B*] were crossed with *w-; UAS-nls:LacZ/TM6B*, *w-; UAS-hFWE2/TM6B*, *w-*; *UAS-dsfoxo*, and *y- w-; UAS-akt*.

To perform the *hFWE* functional conservation studies, we crossed the WT and AD models with controls UAS-nls:*LacZ* and UAS-*fwe^LoseB^*, and UAS-*hFWE1*, UAS-*hFWE2*, UAS-*hFWE3*, and UAS-*hFWE4*. All hFWE stocks had the same background: *y- w- hsFLP; If/CyO; UAS-hFWEx/TM6B* used in Figure 3. The control stocks UAS-nls:*LacZ*, UAS-*fwe^LoseB^* were all *y- w- hsFLP; If/CyO; UAS-X/TM6B.*

### 2.2. Generation of hFWE Constructs

hFWE isoforms were amplified from vectors previously used in [37] and cloned into the pUAStattb vector. Transgenic flies for each isoform were generated by Rainbow Transgenic Flies Inc., Camarillo, CA, USA. hFWE isoform constructs were inserted in *PBac{y[+]-attP-3B}VK00033* (BDSC #24871). For the isoform sequences, see the supplemental data. hFWE3 and hFWE4 are sequences with codon optimisation.

### 2.3. Experimental Protocols

#### 2.3.1. Fly Brain Dissection

For optic lobes, fly brains were dissected in cold PBS 1x, fixed in 3.7% PFA for 30 min at RT, washed with 0.4% PBS-Triton-X (PBS-T), Sigma Aldrich #1002135493, St. Louis, MO, USA for 30 min and incubated with primary antibody (mouse anti-β-Amyloid, 17–24 [4G8] #SIG-39220 1:100 in 0.4% PBS-T and 5% of Normal Donkey Serum Jackson ImmunoResearch #017-000-121, West Grove, PA, USA) overnight at 4 °C. After removing the primary antibody, the tissue was washed for 30 min with PBS-T 0.4%, and incubated with a secondary antibody (Alexa Fluor 488, Invitrogen #A-21202, Carlsbad, CA, USA, 1:1000 in 0.4% PBS-T) at 4 °C overnight. The following day, the secondary antibody was rinsed, and the sample was washed with PBS 1x for 30 min. The adult brains were mounted in Vectashield with DAPI (Vector Labs Inc. #H-1200, Newark, CA, USA) using a spacer to avoid compression of the optic lobes. Samples were imaged on a Zeiss LSM 880 confocal microscope, Oberkochen, Germany.

#### 2.3.2. TUNEL Staining

TUNEL kit assay (Roche #03333566001, Basel, Switzerland) was used with some alterations compared to the manufacturer’s protocol. After brain fixation, brains were washed for 1 h in 0.4% PBS-T and incubated with TdT buffer for 1 h, both at room temperature. Afterwards, samples were incubated in TUNEL solution containing Terminal Transferase enzyme (3 µL/mL) and biotin-16-dUTPs (Roche #11093070910, Basel, Switzerland) (2 µL/mL) diluted in TdT buffer (3 µL/mL) for 2 h at 37 °C. The reaction was stopped with STOP citrate buffer for 15 min at room temperature and then washed in PBS 1x for a total of 4–5 h, with the PBS 1x being replaced every 45 min. Samples were then incubated with Streptavidin Alexa Fluor 488 or 647 conjugated (Invitrogen #S11223, Carlsbad, CA, USA and ThermoFisher S21374, Waltham, MA, USA, respectively) diluted in PBS-T with 10% of Normal Donkey Serum (Jackson ImmunoResearch #017-000-121, West Grove, PA, USA) at 4 °C overnight. The following day, the samples were washed for 2 h with PBS 1x, replacing the PBS every 30 min.

#### 2.3.3. Diets Protocol

Crosses were set up in YBD at a ratio of 3 females to 1 male per vial at 25 °C. After 3 days of egg laying, the flies were discarded, and the progeny were allowed to develop and hatch. After hatching we allowed males and females to be together for 3 days, promoting female mating. On the third day post-hatching, F1 female flies were sorted according to the correct genotype and placed on either a YBD or a synthetic diet for 3, 14, 21, and 28 days. At these time points, we performed locomotion assays and the dissection of fly brains. In the case of the synthetic diet, flies were fed with the exome-matched diet (FLYAA) recipe, developed by Piper et al. (2017) [38], which is the same diet we refer to as the SAA in this study.

#### 2.3.4. Locomotion Assay—Buridan’s Paradigm

Buridan’s paradigm was used to evaluate the locomotion behaviour and was performed as described [39]. Trajectories were analysed using Centroid Trajectory Analysis (CeTrAn) (RRID:SCR_006331) [39] (https://github.com/jcolomb/CeTrAn/releases/tag/v.4, accessed on 7 December 2025) providing eleven metrics to evaluate locomotion. Statistical analysis was performed using GraphPad Prism software 10.

Twenty-four hours before each time point, the wings of mated females were clipped to one-third of their length to prevent them from escaping the arena. The flies were then distributed one per vial and allowed to recover for 24 h. One hour before the assay, flies were transferred to empty vials to prevent grooming behaviour in the arena. Flies were placed individually in the centre of the arena, and their locomotion behaviour was recorded for 5 min using the Buritrack software [39]. The assay was restarted if the flies jumped a maximum of 2 times. Flies immobile for more than 1 min were excluded from the assay. After each run, the flies were dissected. Locomotion assessment was performed after 3, 14, 21, and 28 days of feeding. Flies were flipped every three days to ensure the nutritional quality of the diet.

### 2.4. Image Quantification

We have developed a new quantification method that integrates machine learning with custom MATLAB-based analysis (version R2021b). Ilastik’s Pixel classification workflow was used to categorise pixels into signals of interest based on colour/intensity, edge, and texture [40]. Representative images were uploaded, and the user iteratively annotated the signal(s) of interest (TUNEL and hAβ42—green; *azot*—red) and background, receiving real-time feedback on segmentation accuracy. Once satisfied with the network’s performance, the entire image batch was segmented.

The corresponding probability mask was produced by Ilastik (Figure S6B) indicating the probability that each pixel belonged to the label/signal of interest. A custom MATLAB workflow was used to quantify the segmented signals of interest as follows: merged confocal z-projection images were loaded (Figure S4C), and the optic lobe was manually outlined as the region of interest (ROI). The corresponding probability mask was then loaded, and minimum-size (for TUNEL—17; for hAβ42—18; and for *azot*—17 pixels) and probability thresholds (0.05 for green signal and 0.1 for *azot*) were used to exclude objects that were too small or unlikely to be signals of interest. Consistent size and probability thresholds were applied across all samples.

The image was then binarised, and relevant metrics, such as total area of the ROI, total area of the signal of interest, percentage of overlap of signals of interest, etc., were calculated (Figure S4D–D′). After processing all images, data were exported as Excel files. Data from Excel files were loaded into GraphPad Prism software 10 for graphical representation and statistical analyses.

To use the automated quantification method, we first validated it by comparing it with the ImageJ-Fiji (1.54n) multi-step process, a standard quantification method. We validated the method’s performance through human inspection by comparing the standard and automated methods’ performance. To achieve this, ten images were randomly selected using research randomiser software (Research Randomizer (Version 4.0) [Computer software]. Retrieved on 22 June 2013, from http://www.randomizer.org/). We then produced two images (for the same raw signal): image 1 using Fiji and image 2 using the probability mask created by Ilastik. Figure S4A shows how these images were obtained. Later, we applied the same ROI and the same parameters (thresholding and cell size). This process was performed for all the randomly selected images. Next, we will show the results obtained from all the signals.

Validation of *azot* reporter and TUNEL staining

Based on the presented results (Figure S5), it was clear that signal detection using Ilastik performed as well as, if not better than Fiji Image-J (Figure S5H,I). Thus, given that Ilastik seemed to detect more cells on average than Fiji, we were keen on ensuring that they were not false positives while performing the validation of the method (see Figure S5B–D, where Fiji missed some cells while Ilastik did not). Furthermore, the Ilastik-MATLAB method also allowed us to quantify the amount of *azot*–TUNEL-positive cells since it was designed to detect when two signals colocalised, totally or partially. For quantification purposes, the expression of *azot*, TUNEL, and *azot* area colocalised with TUNEL (*azot*–TUNEL-positive cells) are shown in terms of the area of the signal normalised to ROI.

Aβ42-antibody

Lastly, we also applied this method to quantify the signal obtained for the Aβ42-antibody (Figure S6) in the diet experiments. Ilastik was trained to detect Aβ42-antibody in green. Figure S6I once more shows the better performance of an Ilastik-based method. Apart from the increased sensitivity for signal detection, with appropriate training, Ilastik was also able to eliminate some trachea in the optic lobes, which in ImageJ-Fiji, would be very hard to achieve (Figure S6A–D).

In summary, the new method for quantifying cell competition is highly accurate, reproducible, and reliable, as Ilastik can infer the size, shape, and intensity of the signal. Thanks to its versatility, it enables quick and convenient batch processing of extensive data sets and can be applied to all sorts of experiments.

### 2.5. Statistical Analysis

Our data do not assume a Gaussian distribution, and results are shown as median ± 95% CI. Therefore, the Kruskal–Wallis test was applied with the multiple-comparisons post hoc Dunn’s test. Statistical analyses were performed with GraphPad Prism software 10. Results were considered significant at * *p* < 0.05; ** *p* < 0.01, *** *p* < 0.001, **** *p* < 0.0001.

## 3. Results

### 3.1. Yeast-Based Diet Hinders Cell Competition in AD Model, Leading to Locomotion Decline

Our previous work showed that, at 14 days old, flies expressing *hAβ42* have greater azot expression and cell death than do control flies, and that the activation of cell competition allowed motor improvements in *hAβ42*-expressing flies [13]. We assessed whether diet affects cell competition and *hAβ42* toxicity. Given that in humans AD progresses with age, we decided to include older flies and evaluate *azot*-dependent cell competition and its behavioural implications in the AD model (hAβ42-expressing flies). Moreover, we established a healthy model with one *GFP* copy driven by GMR (*GMR-Gal4*, *UAS-GFP*) to evaluate the dietary effect in the absence of disease. We fed healthy flies (GMR>GFP;azot::mCherry) and hAβ42-expressing flies (GMR>hAβ42;azot::mCherry) for 3, 14, 21, and 28 days on either a Yeast-Based diet (YBD) or Standard Synthetic (SAA) diet, and evaluated unfit cells, cell death, unfit cell elimination, and locomotion. We assessed unfit cells by measuring the area of *azot* expression with an *azot::*mcherry reporter [13] and cell death by the area of TUNEL staining, both in the optic lobe regions where projections of the photoreceptor neurons are present. To evaluate the elimination of unfit cells, we quantified the colocalisation of *azot* with TUNEL staining (which we designated as *azot*–TUNEL-positive cells), using a combination of machine learning and custom-written software, as described in Section 2. Furthermore, we evaluated locomotion through the Buridan paradigm, which was designed to assess visuomotor responses [41].

**Figure 1:**
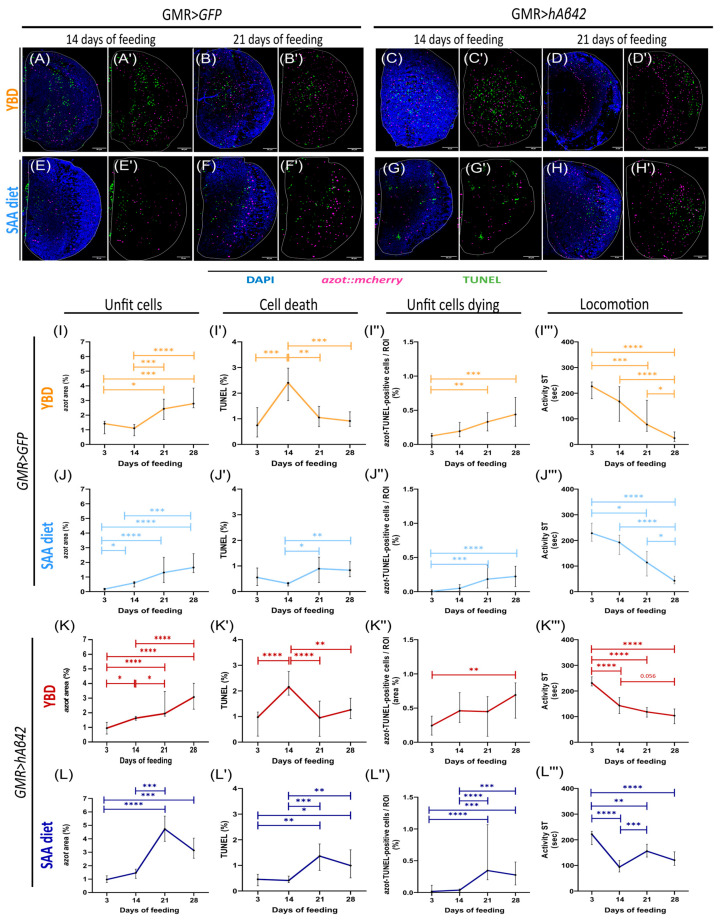
Yeast-based diet (YBD) and Synthetic (SAA) diet have different impacts on cell competition. Representative images of optic lobes (OL) of adult healthy flies (**A**–**B′**,**E**,**E′**), AD model (**C**–**D′**,**G**–**H′**), fed with YBD (**A**–**D′**) and SAA (**E**–**H′**) for 14 and 21 days. Both healthy and AD model flies carry a UAS-LacZ. (**A**–**H′**) DAPI for nuclei label (blue), *azot* reporter (magenta), and TUNEL for cell death label (green). Quantification of *azot* expression shows levels of unfit cells (**I**,**J** for healthy flies; **K**,**L** for AD model). Quantification of TUNEL shows levels of cell death (**I′**,**J′** for healthy flies; **K′**,**L′** for AD model). Quantification of *azot*–TUNEL-positive cells shows levels of unfit cells dying and is measured by area of *azot* and TUNEL colocalising (**I″**,**J″** for healthy flies; **K″**,**L″** for AD model). All quantifications measure the area of the signal divided by the region of interest (ROI), which is the optic lobe (%). Quantification of Activity Speed Threshold represents a measure for a fly’s locomotion and is the amount of time that a fly’s velocity is above a particular threshold—2.7 mm/s (**I‴**,**J‴** for healthy flies; **K‴**,**L‴** for AD model). All flies were fed for 3, 14, 21, and 28 days. For cellular studies (*azot* area, TUNEL area, and area of *azot*–TUNEL-positive cells colocalized), *n* shows the number of optic lobes analysed: (**I**–**I″**) 3d *n* = 13, 14d *n* = 15, 21 d *n* = 17, and 28d *n* = 13; (**J**–**J″**) 3d *n* = 18, 14 d *n* = 25, 21 d *n* = 13, and 28 d *n*= 14; (**K**–**K″**) 3d *n* = 11, 14 d *n* = 42, 21 d *n* = 14, and 28 d *n* = 21; and, (**L**–**L″**) 3 d *n*= 23, 14 d *n*= 40, 21 d *n*= 22, and 28 d *n* = 28. *n* shows the number of flies analysed for locomotion analysis: (**I‴**) 3 d *n* = 35, 14 d *n* = 32, 21 d *n* = 36 and 28 d *n* = 28; (**J‴**) 3 d *n*= 44, 14 d *n* = 39, 21 d *n*= 34, and 28 d *n* = 23; (**K‴**) 3 d *n* = 51, 14 d *n* = 65, 21 d *n* = 39, and 28 d *n* = 36; and, (**L‴**) 3 d *n* = 60, 14 d *n* = 54, 21 d *n* = 59, and 28 d *n* = 60. Results were considered significant at * *p* < 0.05; ** *p* < 0.01, *** *p* < 0.001, **** *p* < 0.0001. Scale = 30 µm.

Healthy flies fed with a YBD (Figure 1A–B′,I–I‴) presented a drastic increase in *azot* expression (Figure 1I) by day 21 of feeding, which remained constant until day 28. A peak in the TUNEL area was detected at day 14 (Figure 1I′), followed by a gradual decline. The area of *azot*–TUNEL-positive cells (Figure 1I″) exhibited a similar pattern to *azot* expression over time, with a significant increase by day 21, which plateaued after that point. These results show an increase in the levels of unfit cells and the same effect in the cells expressing *azot* and TUNEL signal. However, TUNEL levels decreased over time, suggesting impairments in cell elimination. Accordingly, we observed a significant decrease in locomotion at day 21, which continuously decreased up to day 28, which was measured through the activity parameter (Figure 1I‴ and Figure S1A–A‴). Similarly, studies with Canton-S flies have shown that locomotion and climbing behaviours start to decline between 7- and 12-day-old flies [42]. Thus, our findings suggest that the activation of cell competition was not enough to prevent a behavioural decline over time when healthy flies were fed with YBD.

In the AD model flies (GMR>hAβ42;azot::mCherry) fed with the YBD (Figure 1C–D′,K–K‴), we observed a significant increase in *azot* expression (Figure 1K) starting at day 14 of feeding, which continuously increased over time. The TUNEL area (Figure 1K′) exhibited oscillatory levels over time, with a peak at day 14 of feeding, followed by a decrease on day 21 and a tendency to start rising again on day 28, although it was not statistically significant. A similar pattern was obtained for the *azot*–TUNEL-positive cells (Figure 1K″), with a significant increase by day 28 compared to day 3 of feeding. The AD model exhibited a gradual locomotion decline (Figure 1K‴, Figure S1C–C‴) despite the activation of cell competition on day 14, as previously reported by Coelho et al. (2018) [13]. However, over time, the oscillations in cell death levels and a different behaviour from unfit cells and unfit cell elimination levels appear to correlate with the decline in locomotion. The continuous rise in *azot* expression, which is a distinguishable pattern from *azot*–TUNEL-positive cells and TUNEL, suggests that the accumulation of unfit cells may contribute to the deterioration of locomotor function. These results hint that unfit cell elimination (*azot*–TUNEL-positive cells) may be blocked downstream of *azot* activation in flies fed with YBD, and that this diet potentiates hAβ42 toxicity and mobility impairment. Then, we questioned whether these cellular and behavioural effects could be regulated by diet.

### 3.2. Synthetic Diet Delays Cell Competition and Limits Locomotion Decline in the AD Model

To assess the dietary effects of nutrients on cell competition in hAβ42-expressing flies, we fed flies with SAA diet, an exome-matched diet where each nutrient is added separately and in exact concentrations, allowing precise nutrient manipulation [38]. To correlate changes in *azot*-dependent cell elimination and locomotion to the diet, we replicated the previous experiments in YBD using the SAA.

In healthy flies on an SAA diet (Figure 1E–F′,J–J‴), *azot* expression (Figure 1J) increased on day 14. TUNEL levels (Figure 1J′) remained low, increasing from day 21 onwards. After 21 days, flies exhibited a significant increase in *azot*–TUNEL-positive cells (Figure 1J″), which continued to increase on day 28 compared to day 3. Locomotor activity (Figure 1J‴ and Figure S1F–F‴) remained constant until day 14, declining afterwards and reaching significantly lower levels on days 21 and 28 compared to days 3 and 14. Despite the increase in unfit cell elimination by day 21, the locomotion declines at day 14 correlating with the rise in unfit cells at this time point. These results suggest that the increase in unfit cell elimination by day 21 cannot revert the effect of unfit cell accumulation by day 14. Compared with healthy flies fed the YBD (Figure S1E–E‴), SAA diet induced overall lower levels of *azot* expression, being significant at day 3 (Figure 1I–J and Figure S1E). Regarding TUNEL levels (Figure 1I′–J′ and Figure S1E′), SAA diet prevented the peak seen at 14 days with YBD, but by days 21 and 28, both diets induced similar levels. *azot*–TUNEL-positive cells (Figure 1I″–J″ and Figure S1E″) were overall lower with the SAA diet, but statistically significant only on day 14. Locomotion (Figure 1I‴–J‴ and Figure S1E‴) revealed no statistical differences, with both flies exhibiting locomotion decline. The results showed that both diets promote the activation of cell competition, increasing the levels of unfit cells, at 21 days for YBD and 14 days for SAA diet in healthy flies. However, we observed a locomotion decline on both dietary regimens. Thus, the activation of cell competition was not sufficient to prevent ageing-associated locomotion decline. The SAA diet is optimised for development and egg-laying, but this comes at the expense of lifespan [38]. As a result, the decline in locomotion seen on the SAA diet may also be age-related despite activation of cell competition.

When hAβ42-expressing flies were fed an SAA diet (Figure 1G–H′,L–L‴), we observed an increase in the number of *azot*-expressing cells (Figure 1L), TUNEL (Figure 1L′), and *azot*–TUNEL-positive cells (Figure 1L″) by day 21, suggesting that cell competition was induced at 21 days of feeding. In this model, locomotion (Figure 1L‴ and Figure S1H–H‴) tracked the cellular results by decreasing on day 14 and increasing again on day 21. These results suggest that the SAA diet activation of cell competition by day 21 may correlate with recovery of locomotion at day 21. A comparison between diets (Figure S1J–J‴) showed that SAA diet led to a lower *azot* expression than YBD did at days 14 and 28 (Figure 1C–D′,G–H′,K–L and Figure S1J). Furthermore, TUNEL levels (Figure 1K′–L′ and Figure S1J′) were also different, with SAA abolishing the peak seen at day 14 on flies fed with YBD. In terms of *azot*–TUNEL-positive cells (Figure 1K″–L″ and Figure S1J″), hAβ42-expressing flies on YBD showed a peak at day 14, while in SAA-fed flies the increase shifted to day 21. These results indicate that feeding our AD model flies with an SAA diet delayed the activation of cell competition to day 21. These results translated into locomotion with YBD-fed flies being more active on day 14 than SAA-fed flies, while the opposite occurs by day 21 (Figure 1K‴–L‴ and Figure S1J‴). These results highlight the correlation between cell competition activation and locomotor effects. SAA-fed flies improved locomotion on day 21 compared to day 14, whereas YBD-fed flies exhibited a gradual decline over time. Altogether, results show that the YBD and SAA diet have different impacts on *azot*-dependent cell competition modulating the fly’s locomotion. Moreover, YBD induced earlier cell competition than SAA diet but still led to locomotion decline. We then wondered if the effect seen with SAA diet correlates with the levels of hAβ42.

### 3.3. Synthetic Diet Delays the Accumulation of hAβ42

**Figure 2:**
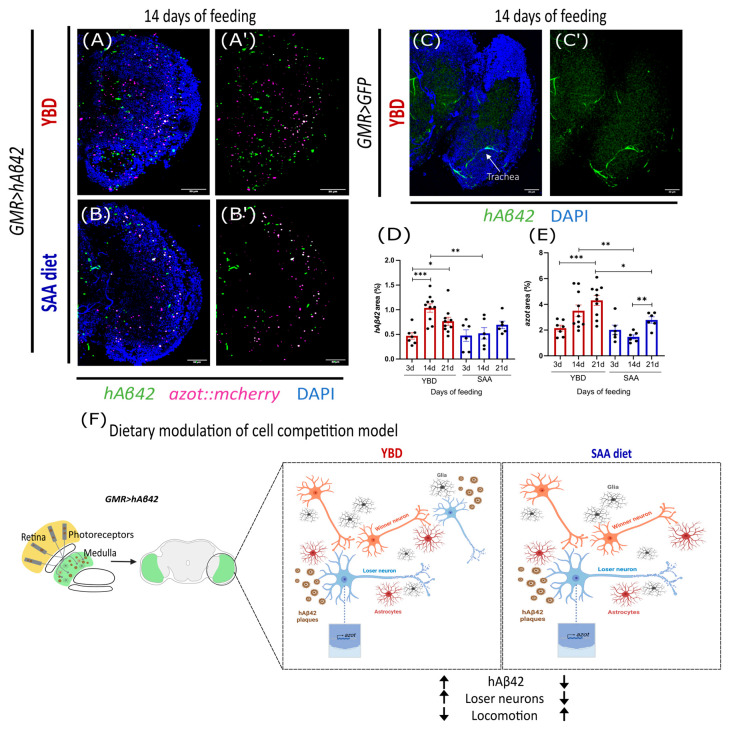
SAA diet prevents high levels of hAβ42 compared to YBD**.** Representative images from the optic lobe of the AD model fed with YBD (**A**,**A′**) and SAA diet (**B**,**B′**) for 14 days, with DAPI (blue), *azot*::mcherry (magenta) and hAβ42 (green). (**C**,**C′**) Representative images of hAβ42 staining with minimal background on healthy flies. (**D**) Quantification of hAβ42 area (%) normalised to the area of the optic lobe. (**E**) Quantification of *azot* area (%) normalised to the area of the optic lobe. Flies were fed for 3, 14, and 21 days. Red bars represent YBD and blue bars represent SAA diet. (**F**) Working Model—Feeding YBD led to higher levels of hAβ42, leading to more loser neurons (blue) and locomotion decline, while SAA diet induced lower levels of hAβ42, less loser neurons, and maintenance of winner neurons (orange), allowing restoration of locomotion upon cell competition. Glia and Astrocytes are represented here as part of the cells in the *Drosophila* optic lobe cells, and no experiments were performed on the cellular types. Image generated with 2025BioRender. Results were considered significant at * *p* < 0.05; ** *p* < 0.01, *** *p* < 0.001. Scale = 30 µm.

We hypothesise that the SAA diet may slow down locomotion decline by limiting hAβ42 accumulation, which may restrain toxicity and the increase in unfit cells, postponing cell competition activation. We fed *hAβ42*-expressing flies with YBD and SAA diet for 3, 14, and 21 days and evaluated the levels of hAβ42 using a specific antibody and measuring the area of signal (Figure 2A–B′,D) and *azot* expression (Figure 2A–B′,E). The hAβ42 antibody showed minimal background on healthy flies at 14 days (Figure 2C–C′), confirming that the signal detected in the AD model represents hAβ42 (Figure 2A–B′). YBD-fed flies increased hAβ42 levels by day 14, which remained higher on day 21 compared to day 3 (Figure 2D), coinciding with the rise in *azot* expression on day 14 (Figure 2E) and confirming our previous *azot* results (Figure 1K). Compared to YBD, SAA-fed flies exhibited approximately half the hAβ42 levels and *azot* signal at day 14 (Figure 2B–B′,D–E). However, by day 21, SAA and YBD-fed flies had comparable levels of hAβ42 (Figure 2D), but SAA-fed flies exhibited lower levels of *azot* expression (Figure 2E), despite being higher than those at day 14 on the SAA diet. To confirm that the observed hAβ42 levels were a direct effect of the diet and not a result of variation in Gal4 system regulation, we assessed GFP intensity using corrected total cell fluorescence in healthy flies fed with both diets (Figure S1K–M). Our results show similar GFP intensities in SAA- and YBD-fed flies, demonstrating that SAA diet has no effect on the Gal4-UAS expression system. Together, the results suggest that the SAA diet may delay the accumulation of hAβ42 protein and *azot*-expressing cells, postponing cell competition to day 21 (Figure S1J–J‴). The lower amount of hAβ42 in SAA-fed flies correlates with the capacity of these flies to recover locomotion on day 21 compared to day 14. In contrast, YBD-fed flies do not have this capacity, and locomotion gradually declines over time (Figure 2E). While we have identified how the SAA diet may be impacting cell competition in the AD model (Figure 2F), it remains uncertain why locomotion is gradually impaired in flies fed with YBD, despite the activation of cell competition at 14 days of feeding.

### 3.4. hFWE Isoforms Are Functionally Conserved in Drosophila Adult Neuronal Tissue

**Figure 3:**
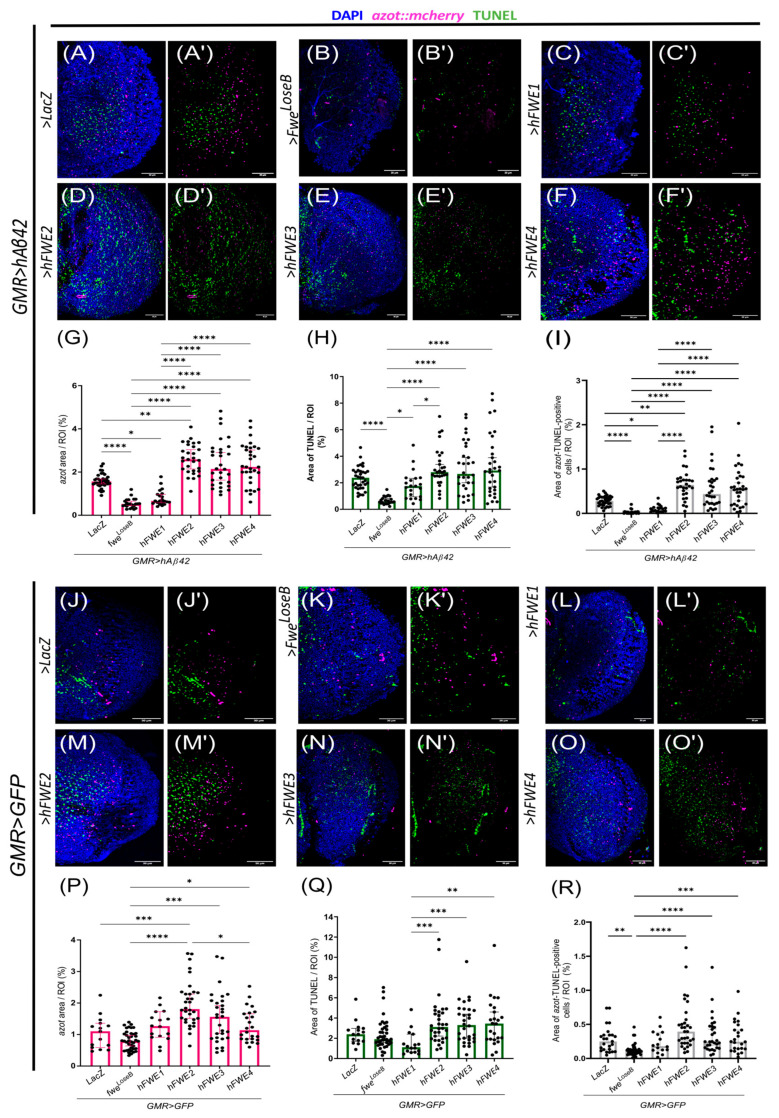
Functional conservation of hFWE isoforms in Drosophila optic lobe. Representative images of optic lobes of flies fed with YBD for 14 days and overexpressing LacZ (**A**,**A′** in AD model, **J**,**J′** in healthy), *fwe^LoseB^* (**B**,**B′** in AD, **K**,**K′** in healthy), *hFWE1* (**C**,**C′** in AD model, **L**,**L′** in healthy), *hFWE2* (**D**,**D′** in AD model, **M**,**M′** in healthy), *hFWE3* (**E**,**E′** in AD model, **N**,**N′** in healthy), and *hFWE4* (**F**,**F′** in AD model, **O**,**O′** in healthy), showing the signals of interest: DAPI (blue), *azot*::mcherry (magenta) and TUNEL (green). Both healthy and AD model flies carry a UAS-LacZ in the absence of *hFWE* expression. (**G**,**P**) Quantification of *azot* expression by *azot*::mcherry reporter signal area normalised to ROI (ROI is the optic lobe) in either AD model or healthy, respectively (%). (**H**–**Q**) Quantification of TUNEL signal area normalised to ROI (%). (**I**–**R**) The quantification of *azot*–TUNEL-positive cells is measured by the area of *azot* reporter signal that is colocalized with the TUNEL signal and normalised to the ROI (%). Our n represents the number of optic lobes, in the AD model LacZ *n* = 35; *fwe^LoseB^* n = 23; hFWE1 *n* = 21; *hFWE2 n* = 29; hFWE3 *n* = 30; hFWE4 *n* = 31; in healthy flies LacZ *n* = 15; *fwe^LoseB^ n* = 39; hFWE1 *n* = 14; *hFWE2* v = 32; hFWE3 *n* = 30; hFWE4 *n* = 24.Results were considered significant at * *p* < 0.05; ** *p* < 0.01, *** *p* < 0.001, **** *p* < 0.0001. Scale = 30 µm.

*hAβ42* expressed under the GMR domain is secreted, and forms aggregates that are extracellular and randomly distributed in the optic lobe [29]. Hence, this AD model generates a heterogeneous environment with neurons of varying fitness status. As demonstrated by Coelho et al. (2018) [13], the neurons near hAβ42 plaques exhibit loser traits, including the expression of *fwe^LoseB^* and *azot*. In this context, the exacerbation of fitness status differences leading to the elimination of loser neurons has beneficial effects, restoring flies’ locomotion [13]. To unravel whether the accumulation of *azot*-expressing cells reflects a blockage of cell elimination, we overexpressed a *fwe* winner isoform to promote fitness differences and force cell competition, potentiating unfit cells’ elimination [13]. We reasoned that if the pathway is not compromised downstream of *azot*, expressing a winner isoform would activate cell competition and potentially restore locomotion. Given that AD is a human disease, we then decided to use a *human Flower (hFWE)* winner isoform to force competition and test our hypothesis. These isoforms were described by our group in the human cancer context, where four *human Flower (hFWE)* isoforms are functionally conserved, with *hFWE1* and *hFWE3* functioning like loser isoforms, while *hFWE2* and *hFWE4* behave as winners [37]. To do so, we first needed to assess the functional conservation of hFWE isoforms in *Drosophila* by overexpressing each isoform individually in the AD model (Figure 3A–I) and healthy (Figure 3J–R) flies. We proceed by assessing unfit cells through *azot* expression, cell death (TUNEL), and the elimination of unfit cells (*azot*–TUNEL-positive cells) at 14 days. In *hAβ42*-expressing flies, the overexpression of a loser hFWE isoform in the photoreceptors surrounded by hAβ42-induced loser cells should decrease cell competition due to increased tissue fitness homogeneity, which would be translated into less *azot* and fewer *azot*–TUNEL-positive cells. In contrast, if an hFWE isoform acts like a winner isoform, it will increase fitness differences between photoreceptors and surrounding tissue containing the hAβ42 plaques. Thus, hFWE winner isoforms should potentiate cell competition, having the opposite effect to hFWE loser isoforms on the amount of *azot* and *azot*–TUNEL-positive cells.

In *hAβ42*-expressing flies, our results show that *hFWE1* (Figure 3C,C′) functions similarly to *fwe^LoseB^* (Figure 3B,B′), hindering competition and functioning as a loser isoform, by decreasing *azot* expression (Figure 3G), TUNEL (Figure 3H), and *azot*–TUNEL-positive cells (Figure 3I) compared to the control (*LacZ*, Figure 3A,A′). In contrast, the overexpression of *hFWE2* (Figure 3D,D′) has the opposite effect on cell competition, inducing an increase in *azot* expression (Figure 3H) and *azot*–TUNEL-positive cells (Figure 3I), thus behaving as a winner isoform. *hFWE3* and *hFWE4* (Figure 3E,E′,F,F′) tend to behave like *hFWE2*, suggesting a winner phenotype, although significant differences occur only when compared to loser isoforms (Figure 3G–I). In healthy flies (Figure 3J–R), *hFWE1* (Figure 3L,L′)- and *fwe^LoseB^*-expressing flies (Figure 3K,K′) show no major differences in *azot* expression, meaning that in a non-sensitised *hAβ42* context, these isoforms are not sufficient to induce cell competition. *hFWE2* significantly increased *azot* expression (Figure 3M,M′,P) compared to the controls, suggesting an increase in fitness differences and thus, cell competition activation. The remaining *hFWE* isoforms (*hFWE3* and *hFWE4*, Figure 3N,N′,O,O′, respectively) had no significant effects on *azot* expression (Figure 3P) and cell death (Figure 3Q) compared with the control LacZ, in a healthy context.

Our results reveal that *hFWE1* acts as a loser isoform, whereas *hFWE3* does not fulfil this role in neuronal tissue, although reported as a loser isoform by Madan et al. (2019) [37]. The distinct behaviour of these human isoforms mirrors the behaviour of *Drosophila* loser isoforms, where *fwe^LoseB^*, like hFwe1, is the only loser isoform in neuronal tissue and functions as a loser in both neuronal and epithelial tissues [25]. On the other hand, *fwe^LoseA^*, as *hFWE3*, does not function as a loser in neuronal tissue [25]. These findings demonstrate the functional conservation of *hFWE* isoforms in *Drosophila* and highlight the role of these isoforms in regulating cell competition. We show that *hFWE1* is the only loser isoform in the neuronal tissue, similar to *fwe^LoseB^*, as previously shown by Rhiner et al. (2010) [24], and that *hFWE2* acts like a winner isoform. *hFWE3* and *hFWE4* have a minor impact on cell competition in a neuronal context, as they are not losers but do not have the same winner behaviour as *hFWE2*. After settling the winner behaviour of *hFWE2* in *Drosophila*, we used this isoform to potentiate competition and evaluated impairments downstream of *azot* in an attempt to unravel YBD effects.

### 3.5. The Effect of hFWE2 Is Diet- and Context-Dependent

To assess whether increasing relative fitness differences enhances unfit cell elimination and prevents locomotion decline in flies fed with YBD, we overexpressed *hFWE2* on healthy and *hAβ42*-expressing flies fed with YBD or SAA diet for 14, 21, and 28 days. We then compared them with the flies without *hFWE2* (control LacZ flies, data from Figure 1) at the same timepoints. *hFWE2* expression should improve unfit cell elimination and prevent locomotion decline, if the *fwe/azot* pathway is not inhibited downstream of *azot* expression. We evaluated the same parameters as before to assess cell competition and locomotion.

**Figure 4:**
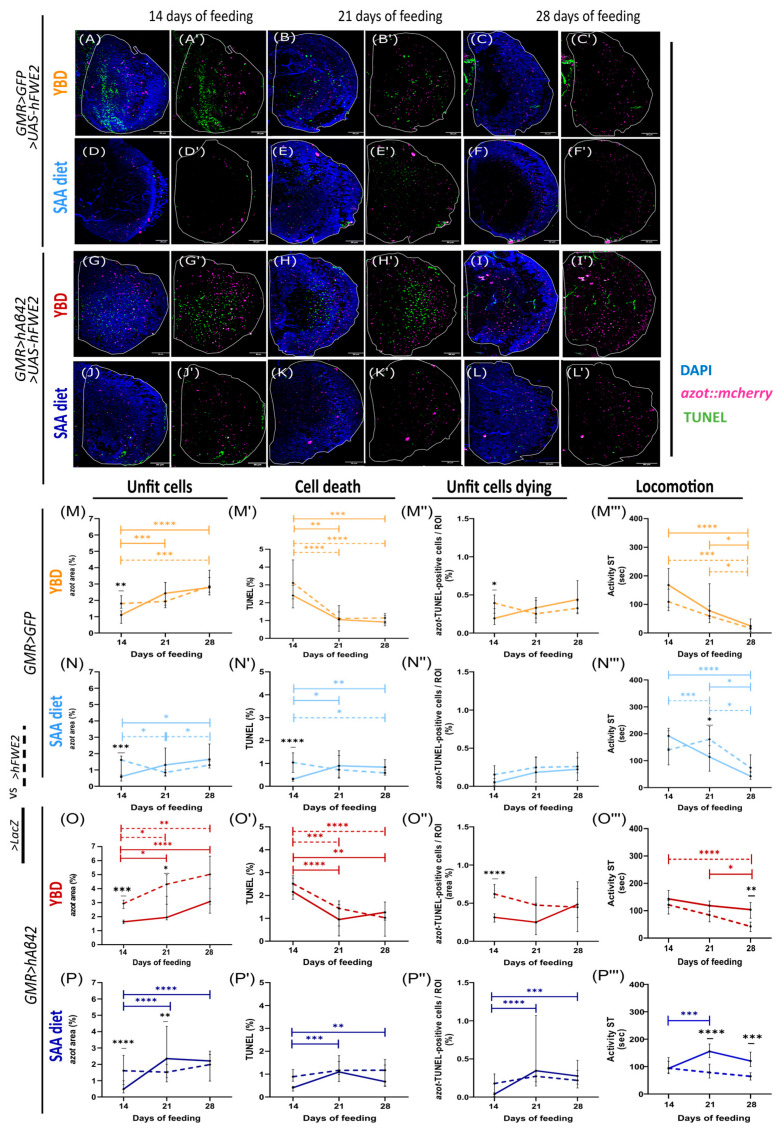
Winner isoform overexpression was not enough to restore locomotion in yeast-based diet. Representative images of optic lobes (OL) of adult healthy flies fed with YBD (**A**–**C′**) and with SAA (**D**–**F′**); and AD model fed with YBD (**G**–**I′**) and SAA (**J**–**L′**) fed for 14 to 28 days. (**A**–**L′**) DAPI for nuclei label (blue), *azot* reporter (magenta), and TUNEL for cell death label (green). (**M**–**P‴**) Dashed lines represent flies expressing *hFWE2* and solid lines represent LacZ-expressing flies (same data from Figure 1). Quantification of *azot* expression shows levels of unfit cells (**M**,**N** for healthy flies; **O**,**P** for AD model). Quantification of TUNEL shows levels of cell death (**M′**,**N′** for healthy flies; **O′**,**P′** for AD model). Quantification of *azot*–TUNEL-positive cells shows levels of unfit cells dying and is measured by area of *azot* and TUNEL colocalising (**M″**,**N″** for healthy flies; **O″**,**P″** for AD model). All quantifications measure the area of signal divided by the region of interest (ROI), which is the optic lobe (%). The quantification of Activity Speed Threshold represents a measure for a fly’s locomotion and is the amount of time that a fly’s velocity is above a particular threshold—2.7 mm/s (**M‴**,**N**,**J‴** for healthy flies; **O‴**,**P‴** for AD model). All flies were fed for 14, 21, and 28 days. For cellular studies (*azot* area, TUNEL area, and area of *azot*–TUNEL-positive cells colocalized) *n* shows the number of optic lobes analysed: (**M**–**M″**) +LacZ at 14 d *n* = 15, at 21 d *n* = 17, and at 28 d *n* = 13; +*hFWE2* flies at 14 d *n*= 32, at 21 d *n* = 18, and at 28 d *n* = 25; (**N**–**N″**) +LacZ flies at 14 d *n* = 25, at 21 d *n* = 13, and at 28 d *n* = 14; +*hFWE2* flies at 14 d *n* = 23, at 21 d *n* = 21, and at 28 d *n* = 30; (**O**–**O″**) +LacZ flies at 14 d *n* = 42, at 21 d *n* = 14, and at 28 d *n* = 21; +*hFWE2* flies at 14 d *n* = 44, at 21 d *n* = 14, and at 28 d *n* = 14; (**P**–**P″**) +LacZ flies at 14 d *n* = 40, at 21 d *n* = 22, and at 28 d *n* = 28; +*hFWE2* flies at 14 d *n* = 18, at 21 d *n* = 33, and at 28 d *n* = 21. In locomotion studies, *n* shows the number of flies analysed: (**M‴**) +LacZ flies at 14 d *n* = 32, at 21 d *n* = 36, and at 28 d *n* = 28; +*hFWE2* flies at 14 d *n* = 27, at 21 d *n* = 30, and at 28 d *n* = 21; (**N‴**) + LacZ flies at 14 d *n* = 39, at 21 d *n* = 34, and at 28 d *n* = 23; +*hFWE2* flies at 14 d *n* = 27, at 21 d *n* = 36, and at 28 d *n* = 39; (**O‴**) +LacZ flies at 14 d *n* = 65, at 21 d *n* = 39, and at 28 d *n* = 36; +*hFWE2* flies at 14 d *n* = 80, at 21 d *n* = 44, and at 28 d *n* = 35; (**P‴**) +LacZ flies at 14 d *n* = 54, at 21 d *n* = 59, and at 28 d *n* = 60; +*hFWE2* flies at 14 d *n* = 53, at 21 d *n* = 57, and at 28 d *n* = 56.Results were considered significant at * *p* < 0.05; ** *p* < 0.01, *** *p* < 0.001, **** *p* < 0.0001. Scale = 30 µm.

In healthy flies fed with YBD and expressing *hFWE2* (Figure 4A–C′, dashed line in M–M‴), *azot* expression increased over time (Figure 4M), while *azot*–TUNEL-positive cells (Figure 4M″) remained stable over time. TUNEL levels gradually decreased (Figure 4M′), with flies exhibiting a locomotion decline over time (Figure 4M‴). Comparing healthy flies expressing *LacZ* (control, solid line, Figure 4M–M‴) with *hFWE2* (dashed line, Figure 4M–M‴), revealed that *hFWE2*-expressing flies exhibit more cell competition at 14 days, being statistically significant for *azot* expression (Figure 4M) and *azot*–TUNEL-positive cells (Figure 4M″). However, *hFWE2* flies exhibited a similar locomotion decline to *LacZ* flies (Figure 4M‴), suggesting that *hFWE2* potentiates age-related locomotion decline on YBD. Our results suggest that when healthy flies are fed with YBD, although *hFWE2* induces a higher activation of cell competition compared to control LacZ at 14 days (Figure 4M–M″), it is not enough to prevent locomotion decline (Figure 4M‴).

In *hAβ42*-expressing flies fed with YBD and expressing *hFWE2* (Figure 4G–I′, dashed line in O–O‴), it was revealed that *azot* expression (Figure 4O) increases, while TUNEL decreases over time (Figure 4O′) and *azot*–TUNEL-positive cells (Figure 4O″) remain stable. This inverse correlation may be responsible for the locomotion worsening in *hFWE2*-expressing flies (Figure 4O‴). These YBD results showed that *hFWE2* is able to increase the levels of unfit cells as seen by the levels of *azot* expression over time (Figure 4O). However, this effect is not translated into a better mobility performance of these flies, leading to a significant locomotion impairment at day 28 (Figure 4O‴). Expression of *hFWE2* in *hAβ42*-expressing flies induced more cell competition at 14 days than the *LacZ*, as seen by *azot* expression and *azot*–TUNEL-positive cells (solid vs. dashed line, respectively, Figure 4O–O″). Thus, *hFWE2* promotes higher fitness differences between photoreceptor neurons and surrounding cells than LacZ at 14 days, but unfit cells are not efficiently eliminated from the tissue, given the increase in *azot* overtime, drastic TUNEL decline, and stabilisation of unfit cell elimination over time (Figure 4O–O″ dashed line), promoting their accumulation and locomotion decline, even more than LacZ flies (Figure 4O–O‴).

We performed the same evaluation in SAA-fed flies. In healthy flies expressing *hFWE2* (Figure 4D–F′, dashed line in Figure 4N–N‴), *azot* expression (Figure 4N) decreased from day 14 to day 21, followed by a rise on day 28 of feeding, highlighting the oscillatory nature of cell competition. TUNEL (Figure 4N′) remained constant until day 28, when it decreased compared with day 14. *azot*–TUNEL-positive cells (Figure 4N″) remained constant until day 28. These flies increased locomotion from day 14 to day 21, and showed a decline from day 21 to day 28. Together, these results suggest that *hFWE2* induced the efficient activation of cell competition by day 14, leading to lower levels of unfit cells in the optic lobe by day 21. These results correlate with improved locomotion by day 21, confirming the correlation between cell competition and locomotion. When comparing *hFWE2*-expressing flies with *LacZ* (Figure 4N–N‴, solid versus dashed line), *hFWE2* induced greater *azot* expression (Figure 4N), TUNEL (Figure 4N′) than LacZ. Moreover, by day 21, *hFWE2* led to better locomotion than LacZ (Figure 4N‴). These results suggest that by day 21, *hFWE2*-expressing flies are healthier, as the majority of unfit cells were eliminated, leading to better locomotion. Analysis of *hFWE2* expression in healthy flies fed SAA diet versus YBD (Figure 4 and Figures S2A–A‴) revealed that SAA-fed flies exhibited less *azot* expression across all time points over time (Figure S2A). Regarding TUNEL (Figure S2A′) and *azot*–TUNEL-positive cells (Figure S2A″), both parameters were significantly lower in SAA-fed flies at day 14. However, over time, *azot*–TUNEL-positive cells were similar in both diets, while the levels of TUNEL were higher in YBD-fed flies by day 28. Healthy flies fed with an SAA diet and expressing *hFWE2* had better locomotion over time than those fed with a YBD (Figure S2A‴). The function of *hFWE2* seems to be modulated by diet. SAA diet promotes an efficient and oscillatory elimination of unfit cells, leading to better locomotion when unfit cells are eliminated from the tissue. Moreover, in YBD-fed flies, *hFWE2* induces cell competition, but there is an accumulation of unfit cells over time, leading to locomotion decline.

Regarding the AD model flies expressing *hFWE2* and fed with SAA diet (Figure 4J–L′, dashed line in Figure 4P–P‴), the levels of *azot* expression (Figure 4P), TUNEL (Figure 4P′), and *azot*–TUNEL-positive cells (Figure 4P″) remained stable over time. Locomotion also remained constant over time (Figure 4P‴). When compared to the control flies expressing *LacZ* (dashed vs. solid line in Figure 4P–P‴), expression of *hFWE2* induced higher levels of *azot* expression on day 14 of feeding (Figure 4P), while TUNEL (Figure 4P′) and *azot*–TUNEL-positive cells (Figure 4P″) were similar to the control. These results show that *hFWE2* expression triggers earlier activation of unfit cells compared to LacZ (Figure 4P–P″), in *hAβ42*-expressing flies fed with SAA diet. Despite this early increase, both genotypes had similar locomotion at day 14 (Figure 4P‴). By day 21 *hFWE2* induced less *azot* than LacZ (Figure 4P dashed line vs. solid line), while the levels of TUNEL and *azot*–TUNEL-positive cells remained similar to the control (Figure 4P′–P″). Locomotion was worse than in the LacZ control (Figure 4P‴). At day 28 all cellular parameters were similar between genotypes (Figure 4P–P″), yet *hFWE2* locomotion remained lower than the control (Figure 4P‴). Taken together, these findings suggest that in *hAβ42*-expressing flies fed with SAA, *hFWE2* expression was not able to improve locomotion compared to the control (Figure 4P‴), despite inducing earlier activation of cell competition than LacZ (Figure 4P–P″), as seen in healthy flies (Figure 4N‴). Thus, suggesting impairments in the elimination of unfit cells in *hFWE2*-expressing flies.

Comparison of *hAβ42*-expressing flies fed with YBD and SAA diet (Figure 4O–P‴ and S2B–B‴) showed that YBD-fed flies expressing *hFWE2* had overall higher *azot* levels (Figure S2B) and *azot*–TUNEL-positive cells, the latter being significant only at days 14 and 21 (Figure S2B″). Regarding cell death, YBD-fed flies presented higher TUNEL levels initially (Figure S2B′), but over time these levels drastically decreased, reaching similar levels to those found in SAA-fed flies on days 21 and 28. Behaviourally, YBD-fed flies exhibited a decline in locomotion, while in SAA-fed flies it remained stable. These results suggest that over time, in YBD-fed flies, there is an accumulation of unfit cells that are not eliminated (Figure 4O and Figure S2B), resulting from the drastic decline in TUNEL and stabilisation of *azot*–TUNEL-positive cells (Figure 4O′,O″), leading to locomotion decline over time. In the SAA diet, *hFWE2* did not induce such high levels of unfit cells and unfit cell elimination as in YBD-fed flies, but over time, locomotion in *hFWE2*-expressing flies was similar in both diets (Figure S2B–B‴). Together, our results show that the effect of *hFWE2* is diet- and context-dependent. In healthy flies fed with SAA, *hFWE2* is able to improve locomotion compared to the control, as a result of efficient cell competition. However, in *hAβ42*-expressing flies fed with SAA, *hFWE2* expression led to worse locomotion than the control. When assessing the effects on YBD-fed flies, *hFWE2* failed to improve locomotion in either healthy or *hAβ42*-expressing flies. Further studies are needed to fully understand these results.

### 3.6. Foxo and Akt Seem to Be Involved in the Regulation of Azot Expression in the AD Model

There is a well-established correlation between AD and insulin signalling (IIS). Several studies suggest that AD may be a degenerative metabolic disease being driven by impairments in brain insulin response [2]. In AD patients, there is aberrant insulin signalling due to inhibition of the pathway downstream of the insulin receptor [2]. The inhibition of protein kinase B (AKT) prevents FOXO from being phosphorylated, allowing its translocation to the nucleus and consequently target gene activation [43]. Given the pivotal role of FOXO in metabolism and cell death [44], we hypothesised that Foxo could regulate *azot* expression. To test our hypothesis, we inhibited *foxo* function by overexpressing either double-stranded RNA (UAS-ds-Foxo) or *akt* (UAS-*akt*), and evaluated the number of *azot*-positive cells in YBD-fed flies at day 14. The results showed that when Foxo function is inhibited also by ds-Foxo (Figure S3B,D) or *akt* (Figure S3C,D), the number of *azot*-positive cells decreases compared to control flies expressing *LacZ* (Figure S3A,D). This result suggests that Foxo regulates *azot* expression at 14 in *hAβ42*-expressing flies fed with YBD, which could be a direct or indirect effect. Moreover, these results offer insight into the potential relationship between IIS, *hAβ42* toxicity, and cell competition. IIS may influence cell competition in our AD model, through the regulation of *azot* expression in a Foxo-dependent manner. Further studies are needed to confirm this mechanism associated with AD.

## 4. Discussion

The prevalence of neurodegenerative diseases such as Alzheimer’s Disease (AD) has increased worldwide, together with life expectancy. However, despite a wider range of studies on the aetiology and risk factors in the last few years, the treatment options focus on symptomatic relief rather than stopping/delaying disease progression [14]. Recently, new approaches have emerged, and dietary patterns have been shown to modulate cognitive decline and prevent disease progression [30]. Cell competition is a surveillance mechanism shown to be involved in AD, with a beneficial effect [13]. Our findings suggest that the timing of *azot*-dependent cell competition activation in the AD fly model is diet-dependent (Figure 1). We observed a correlation between cell competition activation with efficient elimination of unfit cells and locomotion improvements (Figure 1 and Figure 4). Our results showed that diet modulates these events. A synthetic diet has the potential to delay unfit cell elimination, with locomotion being improved when cells are efficiently eliminated. Our findings also show that a synthetic diet regulates hAβ42 toxicity by delaying the accumulation of hAβ42 protein (Figure 2).

### 4.1. Yeast-Based Diet Leads to Locomotion Decline Despite Cell Competition Activation

In *hAβ42*-expressing flies fed a yeast-based diet (YBD) (Figure 1K–K‴), *azot*-dependent cell competition was induced earlier than in those on a synthetic (SAA) diet (Figure 1L–L‴). Despite *azot* activation at 14 days, unfit cells continued to rise (Figure 1K), while unfit cells dying followed an oscillatory pattern (Figure 1K″). These results are consistent with the persistence of unfit cells in the tissue and correlate with the locomotion decline over time (Figure 1K‴). Given prior studies showing that cell competition benefits *hAβ42*-expressing flies by improving their locomotion [13], we hypothesised that YBD may impair *azot*-dependent cell competition downstream of *azot*, allowing unfit cells to persist (Figure 2F). Previous research on YBD-fed flies revealed a cyclical unfit cell accumulation and elimination in *azot^−/−^* flies, suggesting that an *azot*-independent mechanism removes suboptimal cells when *Fwe*-mediated cell competition is compromised [28]. In flies with intact cell competition, less than 50% of loser cells expressing *fwe^LoseB^*, activate *azot* and undergo apoptosis, hinting that unfit cells are not always eliminated [28]. Researchers proposed that other parallel pathways may counteract the effect of *fwe^LoseB^*, challenging *azot*’s role as the ultimate fitness sensor [28]. Different studies suggest that *azot* integrates signals from multiple factors, including relative levels of Fwe^lose/win^; Sparc levels, and the percentage of winner neighbouring cells [23,26,45]. *Sparc*, the *Drosophila* homolog of SPARC/Osteonectin family, is upregulated in loser cells, counteracting *fwe* effects and preventing apoptosis during Fwe-independent cell competition [23]. It is plausible that Sparc expression increases in YBD-fed flies, hampering unfit cell removal downstream of *azot*.

In accordance, high levels of SPARC-like 1 were detected in AD patients’ cerebrospinal fluid, proposing SPARC as a potential biomarker [46]. In mice neural stem cells, Testican2 is regulated by Brd4 (Bromodomain Containing 4) and is responsible for Sparc degradation, regulating cell competition, and ensuring the elimination of unfit cells [47]. Additionally, dementia patients exhibited higher levels of Sparc and low levels of Brd4, and mice carrying the Brd4 mutations that are present in these patients show impairments in neural stem cell competition [47]. Reduced mRNA levels of winner isoforms m*Fwe*3 and m*Fwe*4 were found in the cortex of *Brd4*KO aged mice, where Testican2 does not degrade Sparc. These findings indicate a reduced capacity for cell competition and raise the possibility for Sparc to regulate Flower in mammals [47]. Although Portela et al. (2010) stated that *Fwe* and Sparc act in parallel pathways in the epithelial imaginal disc, neuron-specific mechanisms may differ due to the importance of neurons to the fly’s visual system [23]. In our AD model, a similar process may be at play: the silencing of Fs(1)h (female sterile (1) homeotic), the *Drosophila* ortholog of Brd4, reduces cell death in the same *Drosophila* AD model [48]. We speculate that Fs(1)h promotes Sparc degradation, enabling *azot*-dependent elimination of unfit cells up to a threshold, beyond which further cell loss becomes detrimental. However, YBD may induce excessive stress either metabolically or due to higher levels of hAβ42 accumulation than the SAA diet (Figure 2D), which disrupts this balance, mimicking the Brd4 mutation effect in patients, activating Sparc and preventing cell death. YBD thus appears to impair the efficiency of unfit cell elimination, and counteract Fwe’s effects, possibly as a protective mechanism to prevent excessive elimination of important cells involved visual network.

Cell functions rely on nutrients provided by the diet. Thus, nutritional composition may influence the balance between the *Fwe* pathway and parallel pathways that prevent unfit cell removal. Cellular stress levels can favour one pathway instead of the other. In humans, insulin, IGF-1, or LEPTIN are SPARC stimulators, showing that nutrient-sensing pathways can modulate SPARC [49]. The ability of nutrient-sensing pathways to regulate cell competition regulators supports our findings that efficient elimination of unfit cells is diet-dependent. Based on this, we wondered whether some key metabolic players could be modulating *azot* expression. In *hAβ42*-expressing flies fed with YBD, downregulation of *foxo* or overexpression of *akt* was able to downregulate *azot* levels compared to control. Both genes are involved in the IIS pathway, which is compromised in AD [2]. When IIS is impaired, Akt is unable to phosphorylate Foxo, allowing its nuclear translocation and activation of target genes [2]. Since the restriction of Foxo function limits *azot* expression, there is the possibility of *azot* being a Foxo-target gene. Nevertheless, our results cannot discriminate the nature of this effect, whether it is direct or indirect, but are consistent with the perspective that a cell with impaired energetic metabolism will exhibit decreased fitness, thereby fostering a competitive phenotype. *foxo* has previously been implicated in cell competition, with *Foxo3* being upregulated in loser cells due to stressful conditions [50]. To address impairments downstream of *azot* in YBD-fed *hAβ42*-expressing flies, we overexpressed an *hFWE* winner isoform in the GMR domain (dashed lines in Figure 4), thereby intensifying cell competition between photoreceptors and surrounding cells. *hFWE2* increased *azot* expression over time, while cell death, elimination of unfit cells, and locomotion gradually declined, similarly to the control LacZ (Figure 4O′–O‴). These results are in accordance with the idea that an unidentified player(s) is preventing the efficient elimination of unfit cells over time, blocking the pathway. Thus, promoting fitness differences with *hFWE2*, when the pathway is blocked, is detrimental and leads to locomotion decline. Our study also reveals that hFWE isoforms are functionally conserved in *Drosophila* adult neuronal tissues (Figure 3), as *hFWE1* acts like a loser isoform and *hFWE2* acts like a winner isoform. *hFWE3* and *hFWE4* are significantly different from loser isoforms and similar to *hFWE2*, but do not induce more cell competition than the control LacZ. These results support the idea/fact that the hFWE isoforms, as *Drosophila* isoforms [25], behave in a cell-type specific manner. In neuronal tissues, *hFWE2* is a winner isoform, while *hFWE3* and *hFWE4* do not have such a winner or loser phenotype. This work shows the cell-type specific function of hFWE isoforms for the first time in neuronal tissues, given that Madan et al. (2019) [37] described *hFWE1* and *hFWE3* as loser isoforms and *hFWE2* and *hFWE4* as winner isoforms in epithelial cancer cells. Furthermore, Petrova et al. (2012) showed that clonal overexpression of the four mouse Fwe (mFwe1, mFwe2, mFwe3, mFwe4) isoforms in WID led to the elimination of clones expressing the mFwe1 and mFwe3 loser isoforms, while the mFwe2 and mFwe4 winner isoforms had no effects on clone elimination [51]. However, during skin papilloma formation in mice, the levels of mFwe1 increased in the surrounding cells, and those from mFwe2 increased in cancer cells, while mFwe3 and mFwe4 levels showed no significant effects. Therefore, it is not surprising that the expression of hFWE3 and hFWE4 did not lead to outcomes identical to those of *hFWE2*. Expressing hFWE alongside *hAβ42* enhances the humanisation of this AD model, improving studies on the AD–cell competition link.

### 4.2. Synthetic Diet Delays hAβ42 Formation and Cell Competition Activation in AD Model

*hAβ42*-expressing flies fed with SAA diet (Figure 1L–L‴) exhibited an increase in *azot*, TUNEL, and *azot*–TUNEL-positive cells only at 21 days, later than the 14 days from those on YBD (Figure 1K–K‴). Furthermore, at 21 days we observed an increase in locomotion compared to day 14 (Figure 1L–L‴) in SAA-fed flies, while in YBD-fed flies, locomotion declines over time despite competition activation at day 14 (Figure 1K‴). The SAA diet benefits *hAβ42*-expressing flies by efficiently eliminating unfit cells and allowing an increase in motor activity when cell competition is triggered (Figure 1L–L‴). Thus, SAA seems to delay *hAβ42* accumulation in our AD *Drosophila* model, allowing flies to recover their locomotion after 21 days of feeding. Moreover, SAA diet reduces *hAβ42* levels at 14 days compared to YBD-fed flies (Figure 2D), explaining the later activation of cell competition. Together, these findings align with the evidence that diets rich in fruits, vegetables, fish, legumes, and unsaturated oils can reduce Aβ levels in humans (as reviewed in [52]). In AD brains, insulin signalling dysregulations cause insulin resistance and decrease insulin-degrading enzyme (IDE). Since IDE also degrades Aβ, these metabolic changes in AD brains promote Aβ accumulation and plaque formation [2]. It is expected that diets that alleviate the burden of insulin resistance and metabolic stress will promote Aβ clearance, delaying AD progression. We speculate that SAA is a more balanced diet than YBD and may reduce the Aβ similarly, as IDE is conserved in *Drosophila* and mitigates Aβ neurotoxicity [53].

When we expressed *hFWE2* in the AD model flies fed with SAA, although the levels of unfit cells were higher than the control LacZ at 14 days, the cell competition mechanism seems to stabilise over time (Figure 4P–P″), leading to worse locomotion than the control flies (Figure 4P‴). *hFWE2*-expressing flies exhibited similar locomotion phenotypes in either YBD or SAA diet (Figure S2B″), suggesting that *hFWE2* was not able to induce efficient cell competition mechanisms in *hAβ42*-expressing flies fed with both diets. We hypothesise that there is a threshold until which cell competition is beneficial, and when it is surpassed, such as in AD flies expressing *hFWE2,* the mechanism is downregulated to prevent excessive cell death. Our results suggest that diet modulates the elimination of unfit cells in *hAβ42*-expressing flies and that locomotion correlates with cell competition efficiency in a context-dependent manner. Nevertheless, we cannot exclude that locomotion phenotypes may be partially due to GMR-Gal4, since it is a driver known to induce toxicity and retinal degeneration [54,55]. We evaluated locomotion through the Buridan paradigm, which was designed to assess visuomotor responses [41]. Given that photoreceptor neurons, as part of the GMR domain, directly feed into circuits in the central brain responsible for the motor behaviour of the legs and wings, we believe that this paradigm is a valid option to assess the locomotor effects [56]. Given that different outcomes were seen in SAA- and YBD-fed flies (Figure 1L–L‴), this is an indication that locomotion effects may be related to efficient cell competition and diet. Thus, the SAA diet might be sufficient to overcome the GMR effect. Altogether, our results show that YBD seems to prevent efficient elimination of unfit cells, leading to locomotion decline. In contrast, in AD flies, SAA diet delayed the need to activate cell competition, as it prevents the increase in the levels of hAβ42. Further studies are needed to understand how specific nutrients provided by diet and molecular signals relate to competition and their impact on ageing and neurodegenerative diseases.

## 5. Conclusions

Our study provides valuable insights into dietary influence on both the efficiency and timing of cell competition mechanisms, with a direct consequence for disease outcome. While the precise underlying molecular processes remain to be fully elucidated, our findings demonstrate a functional link between nutrition, cell competition, and neurodegeneration. Moreover, we demonstrate hFWE-functional conservation in healthy and AD models. Further studies should clarify the molecular mechanism that modulates *azot*-dependent elimination and *hFWE2* function, investigate the compensatory effects of parallel pathways like Sparc, and determine whether there are context-dependent limits to the removal of unfit cells. Overall, our work advances the understanding of diet-driven effects on cell competition in healthy and hAβ42-expressing flies, highlighting nutritional modulation as a promising strategy to slow neurodegenerative disease via conserved fitness-sensing pathways. This work lays the groundwork for future studies in the field to investigate how fine-tuning cell competition and metabolic health could be a therapeutic strategy in NDDs. We believe our findings help bridge the gap in the field and shed light on new potential strategies for delaying AD progression.

## Data Availability

All relevant data and details of resources can be found within the article and its Supplementary Information. The code generated for quantifications is available upon request.

## Supplementary Materials

The following supporting information can be downloaded at: https://www.mdpi.com/article/10.3390/cells14242011/s1, Figure S1. Effects of Yeast-based diet compared to the SAA diet; Figure S2. Comparison of hFWE2-expressing flies fed with SAA vs YBD; Figure S3. azot expression regulation by key metabolic regulators; Figure S4. Workflow followed to validate the method; Figure S5. Validation of Azot and TUNEL signals for the Ilastik-based quantification method; Figure S6. Validation of Aβ42 signal for the Ilastik-based quantification method.
