# Supplementary material for: Dietary Interventions Modulate Cell Competition and Locomotor Decline in an Alzheimer’s Disease Drosophila Model
Source: Cells. 2025 Dec 17;14(24):2011. doi: 10.3390/cells14242011 (PMC12731619; doi:10.3390/cells14242011)
Supplement: Supplementary file 1 [file cells-14-02011-s001.zip › cells-3840207-supplementary.pdf]

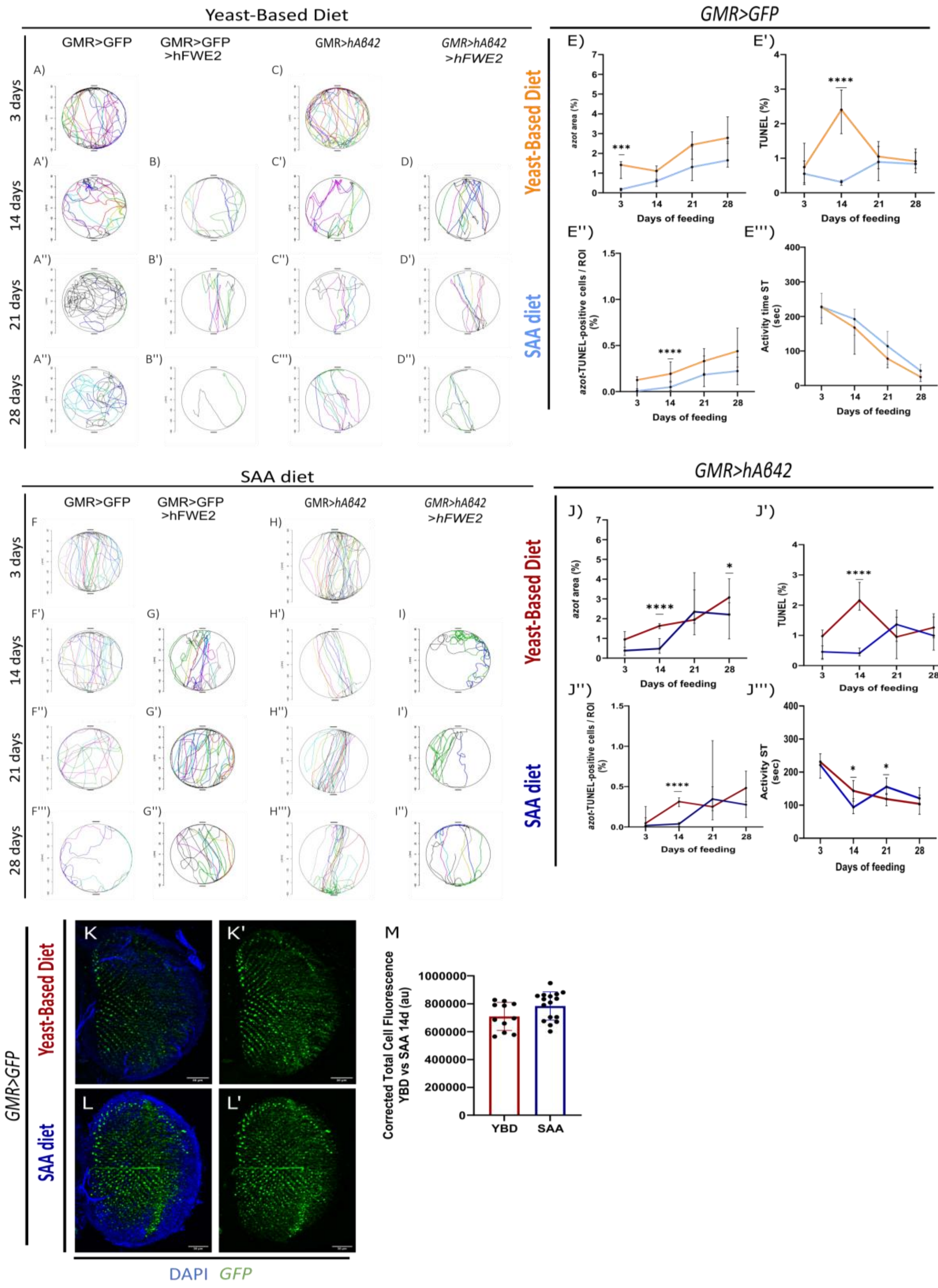

Figure S1 - Effects of Yeast-based diet compared to the SAA diet – (A-D’’) Locomotion data of flies fed with YBD. (E-E’’’) Data from control flies *(GMR>GFP)* fed with YBD (orange) or SAA diet (light blue). (F-I’’) Locomotion data of flies fed with SAA diet**.** (J-J’’’) Data from AD flies (GMR>hAβ42*)* fed with YBD (red) and SAA diet (dark blue). (E,J) Area of *azot* normalized to the ROI (%). (E’,J’) Area of TUNEL normalized to the ROI (%). (E’’,J’’) azot-TUNEL-positive cells is the *azot* area colocalized with TUNEL area (%). (E’’’,J’’’) Fly’s activity in seconds. All flies were fed for 3, 14, 21, and 28 days in the corresponding diet*.* Data presented in A-J’’’ is the same shown individually in Fig1. (K-M) Quantification of GFP intensity in the GMR domain of control flies *(GMR>GFP)* fed for 14 days in either YBD or SAA diets. (K-K’) Representative image of optic lobe from flies fed with YBD. (L-L’) Representative image of optic lobe from flies fed with SAA. (M) Corrected Total Cell Fluorescence in flies fed with YBD (red) and SAA (blue). ROI is the optic lobe.

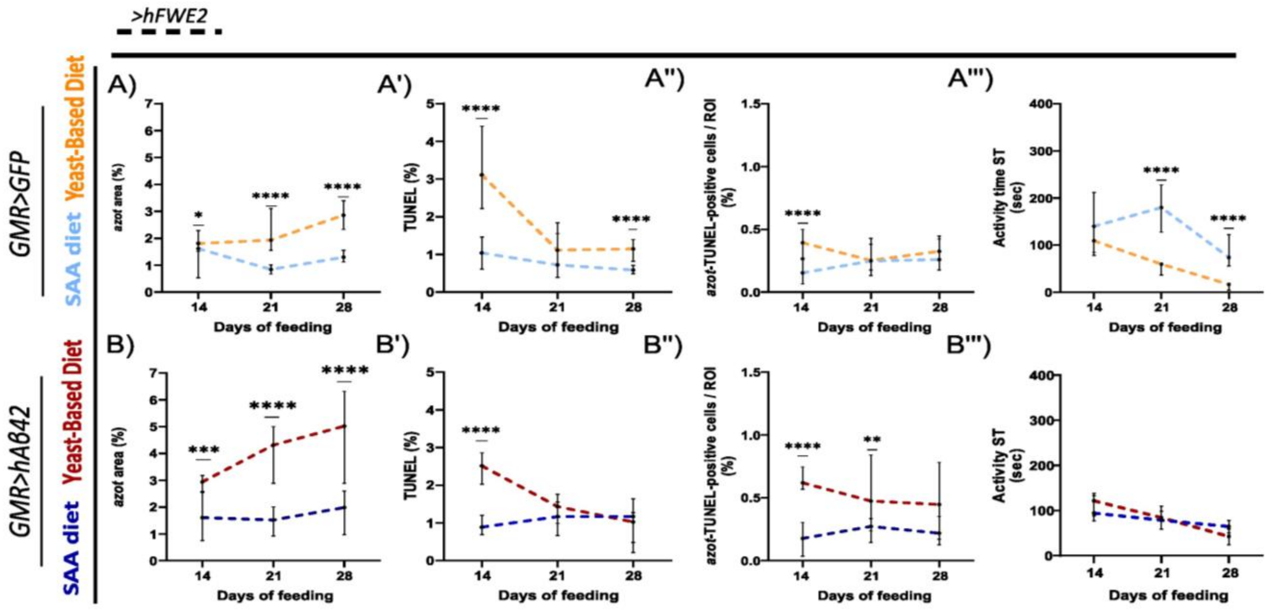

Figure S2 – Comparison of hFWE2-expressing flies fed with SAA vs YBD. (A-A''') Control flies (*GMR>GFP*) expressing hFWE2 and fed with YBD (orange) and SAA diet (light blue). (B-B''') AD flies (*GMR>hAβ42*) expressing hFWE2 and fed with YBD (red) and SAA diet (dark blue). (A, B) Area of azot normalized to ROI (%). (A',B') Area of TUNEL normalized to ROI (%). (A'',B'') azot-TUNEL-positive cells is the azot area colocalized with TUNEL area normalized to ROI (%). (A''',B''') Fly's activity in seconds. All flies were fed for 14, 21, and 28 days in the corresponding diet. ROI is the optic lobe. Data presented here is the same individually shown on Fig4 by the dashed lines.

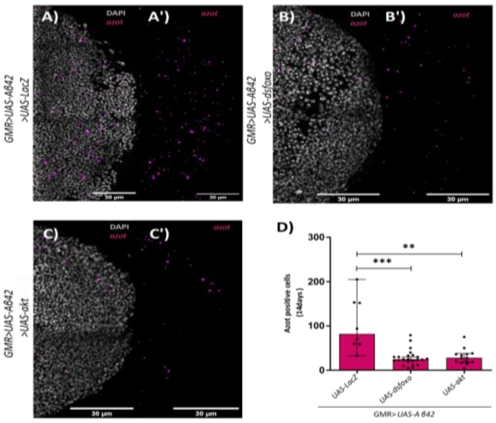

Figure S3 –*azot* expression regulation by key metabolic regulators. *azot* expression (magenta) in AD flies (*GMR>hAβ42)* when *LacZ (*control, A, A'), *ds-foxo* (B, B') or *akt* (C, C') were expressed. DAPI signal (grey). D) Quantification of the *azot* levels in A-C.

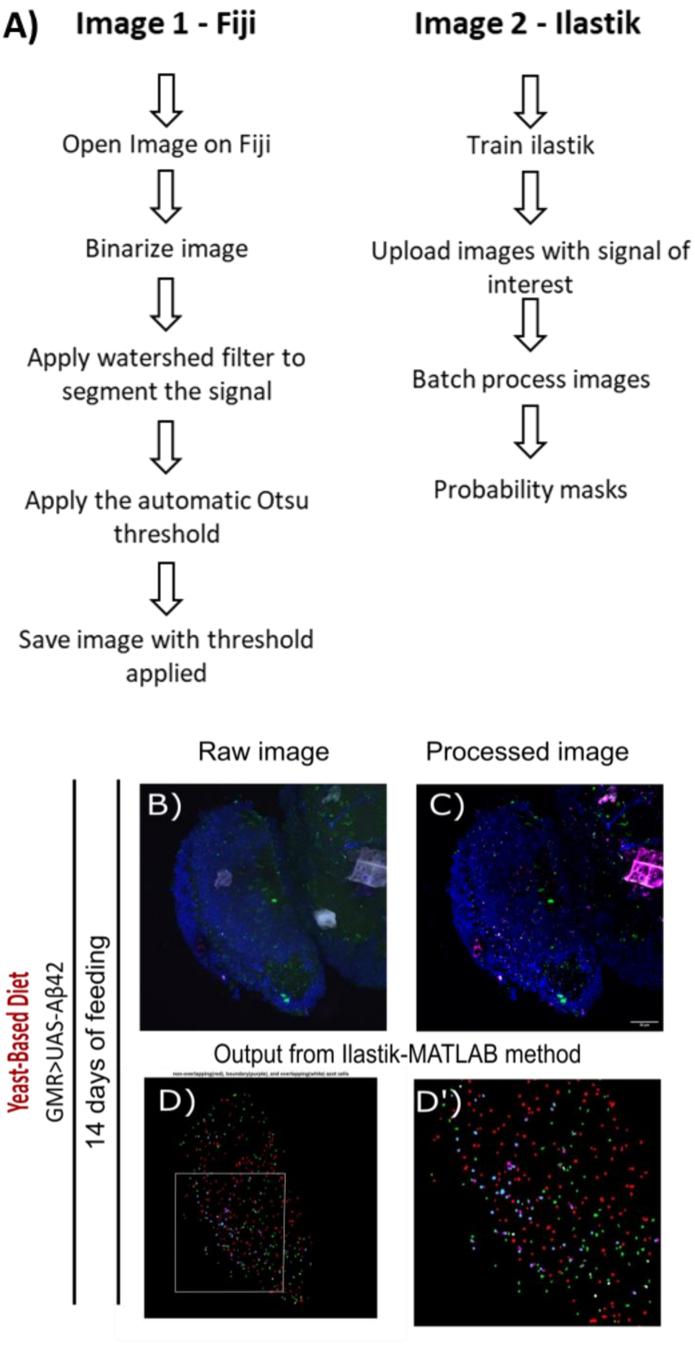

Figure S4- Workflow followed to validate the method. A) Image 1 is the final image obtained to detect the signal of interest with Fiji approach (see FigS5 C,F and FigS6G). Image 2 is the image obtained from signal detection through Ilastik (see FigS6B) which will be subject to MATLAB threshold and quantifications (see FigS5D, G and FigS6 H). For each of the ten images randomly analysed, we did these two images and quantified them, to validate the method. B-D') Sequence of images from raw data to output image with the quantified data through the method developed. B) Raw microscopy image from AD model flies fed with YBD for 14days signal (green – Aβ42, magenta-*azot* reporter, blue-DAPI). C) Z-stack in confocal image B after background removal and to ensure consistency across the manuscript, we standardized the orientation of the optic lobes to facilitate visual comparison; we also removed a pink artefact from image B. D) Output image obtained with the Ilastik-MATLAB method showing the quantified signal (green – Aβ42, red-*azot* reporter, pink/blue – *azot*-TUNEL-positive cells that colocalize). D') Zoom in from D image. The Otsu threshold method minimises the intra-class intensity variance, which measures how similar or different the pixels are within a group. This method aims to find a threshold that minimises this variance when splitting the pixels into two groups (signal and background). After testing several methods, this one reproduced the original signal more accurately.

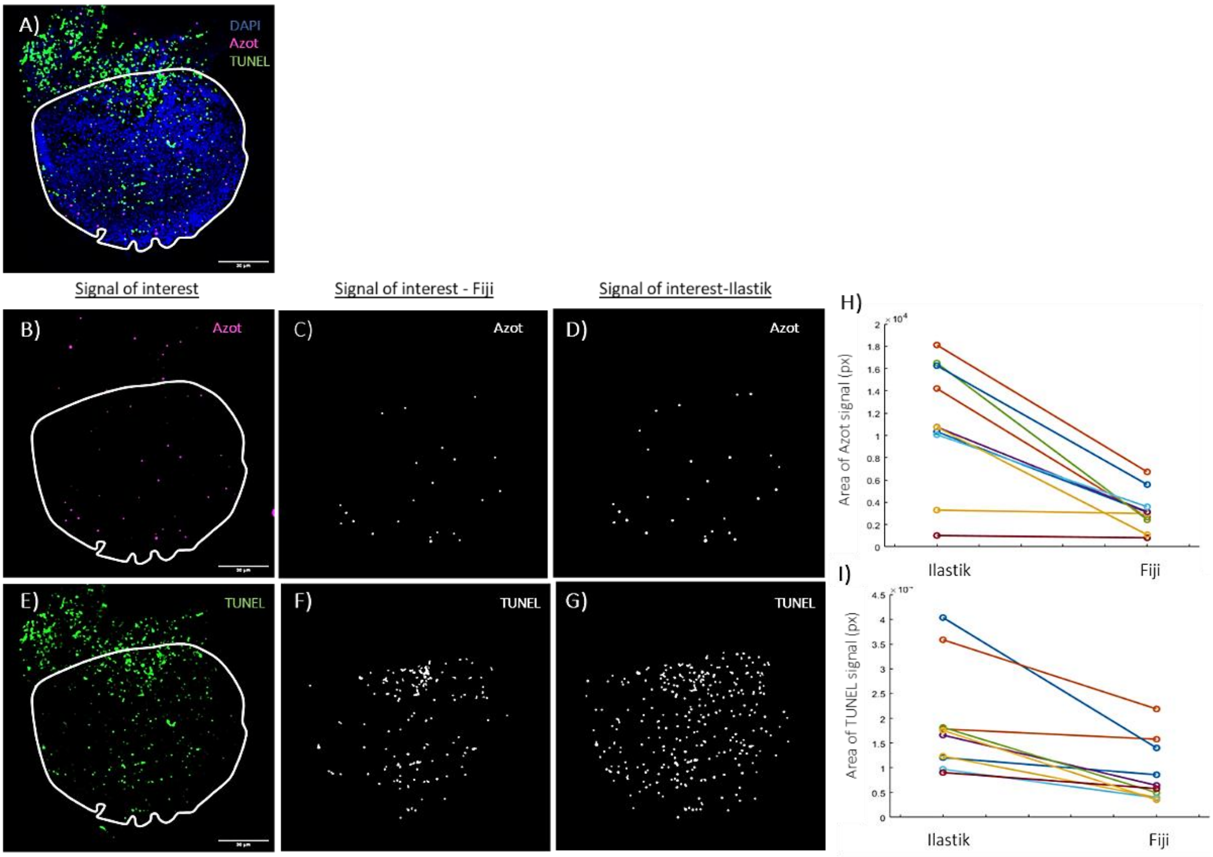

Figure S5- Validation of *Azot* and TUNEL signals for the Ilastik-based quantification method. A) Z-stack with the ROI in the optic lobe, showing in blue DAPI staining, magenta *azot* expression, and green TUNEL staining. B) Raw *azot* reporter signal. C) *azot* reporter signal detected after loading Fiji image (Image1 from FigS4) into MATLAB and applied thresholds. D) *azot* reporter signal detected loading Ilastik image (Image2 from FigS4) into MATLAB and applied thresholds. E) Raw TUNEL signal. F) TUNEL signal detected after loading Fiji image into MATLAB and applied thresholds. G) TUNEL signal detected loading Ilastik image into MATLAB and applied thresholds. H) Graphic showing the area of *Azot* signal in the ROI obtained with both methods in pixels. I) Graphic showing the area of TUNEL signal

in the ROI obtained with both methods in pixels. Each line represents one random image evaluated**.**

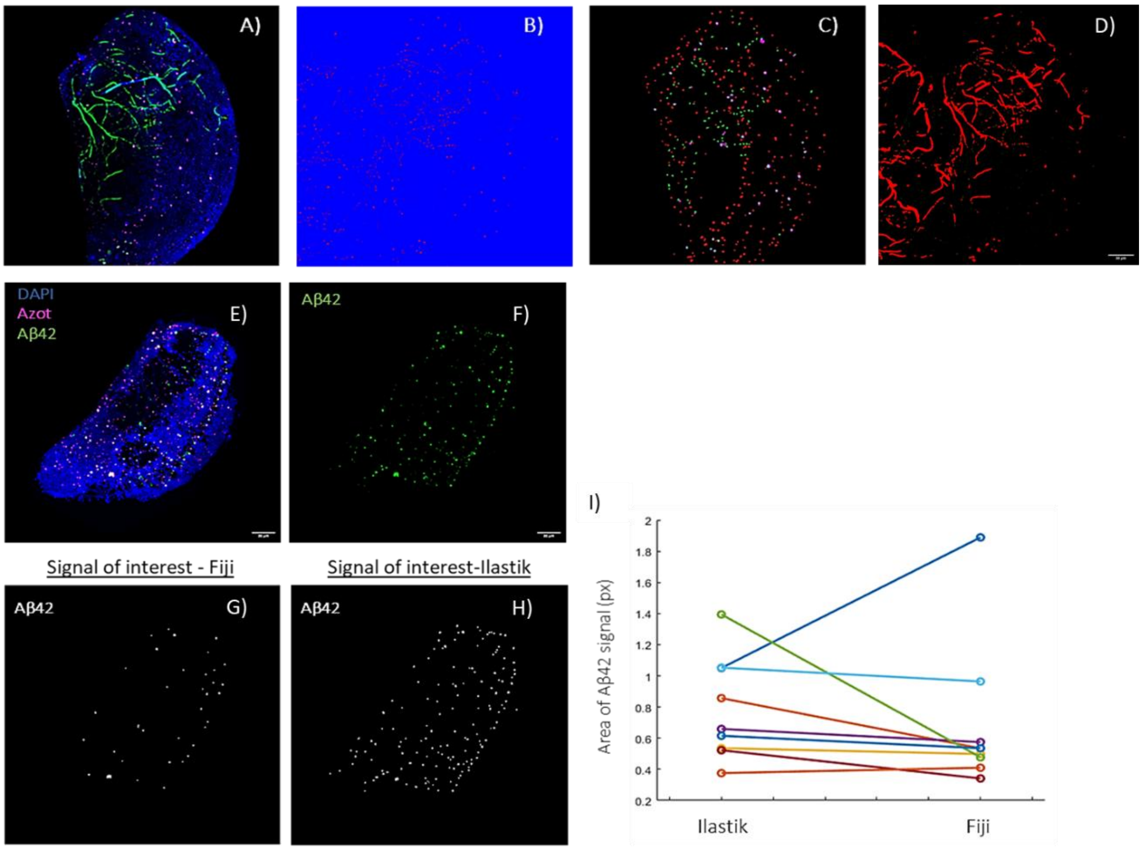

Figure S6 - Validation of Aβ42 signal for the Ilastik-based quantification method. A) Original image with trachea. B) Probability mask showing only Aβ42 signal (Image2 from FigS4). C) MATLAB output with the signal of Aβ42 in green, *azot* that not colocalise in red and azot that colocalises with Aβ42 in pink. D) Output from Fiji after threshold was applied to the green channel**.** E) Z-stack with the region of interest in the optic lobe, showing in blue DAPI staining, in magenta *azot* expression and in green the presence of Aβ42 plaques. F) Raw Aβ42 signal. G) Aβ42 signal detected and quantified after size and threshold upon the Fiji image was loaded on the MATLAB script. H) The Aβ42 signal detected and quantified upon the Ilastik probability mask was loaded into the MATLAB script. I) Graphic showing the percentage of Aβ42 signal obtained with both methods, based on Ilastik and Fiji. Each line represents one random image evaluated.
